# Efficacy and Potential Mechanisms of Naringin in Atopic Dermatitis

**DOI:** 10.3390/ijms252011064

**Published:** 2024-10-15

**Authors:** Seung-Ah Yoo, Ki-Chan Kim, Ji-Hyun Lee

**Affiliations:** 1 Department of Dermatology, Seoul St. Mary’s Hospital, College of Medicine, The Catholic University of Korea, Seoul 06591, Republic of Korea; useunga@catholic.ac.kr; 2 Department of Medical Sciences, Graduate School of The Catholic University of Korea, Seoul 06591, Republic of Korea; kimkichan01@naver.com

**Keywords:** atopic dermatitis, flavonoids, inflammation, naringin

## Abstract

Atopic dermatitis (AD) is one of the most prevalent chronic inflammatory skin diseases. Topical treatments are recommended for all patients regardless of severity, making it essential to develop an effective topical AD treatment with minimal side effects; We investigated the efficacy of topical application of naringin in AD and explored the possible mechanisms using an AD mouse model induced by 1-chloro-2,4-dinitrobenzene (DNCB). Clinical, histological, and immunological changes related to AD and Janus kinase (*JAK*)-signal transducer and activator of transcription (*STAT*) signaling proteins in the skin tissues were measured as outcomes; Naringin treatment resulted in a significant improvement in dermatitis severity score and reduced epidermal thickness and mast cell count in the skin (*p* < 0.05). Naringin also demonstrated the ability to inhibit DNCB-induced changes in *interleukin* (*IL*) 4, *chemokine (C-C motif) ligand* (*CCL*) 17, *CCL22*, *IL1β*, interferon-gamma (*IFN-γ*), and tumor necrosis factor-alpha (*TNF-α*) levels by quantitative real-time polymerase chain reaction (qRT-PCR) and *IL13* by enzyme-linked immunosorbent assay (ELISA) (*p* < 0.05). Western blot results exhibited the decreased *JAK1*, *JAK2*, *STAT1*, *STAT3*, *phospho-STAT3*, and *STAT6* expression in the naringin-treated groups (*p* < 0.05); The findings of this study suggest that topical naringin may effectively improve the symptoms of AD and could be used as a therapeutic agent for AD.

## 1. Introduction

Atopic dermatitis (AD) is a burdensome chronic skin disease, with a worldwide prevalence of up to 20% in the pediatric population and 10% in adults [1,2,3]. The pathogenesis of AD is marked by immune dysregulation, primarily driven by T helper (Th) 2 cytokines such as interleukin (IL) 4, IL5, IL13, leading to inflammatory responses. Acute AD is Th2-dominant, whereas chronic AD shifts to a mixed Th1/Th2 response, with Th1 cytokines like interferon-gamma (IFN-γ) sustaining inflammation [4]. These processes contribute to persistent and severe pruritus and sleep disturbances, imposing a significant disease burden on affected individuals [5]. Current treatment guidelines for AD recommend the use of topical corticosteroids (TCS) and topical calcineurin inhibitors (TCI) for all patients, regardless of severity [6,7]. Both effectively reduce inflammation and pruritus but are limited by adverse effects. TCS cause skin atrophy and pose systemic absorption risks, while TCI often lead to burning and discomfort in nearly 50% of patients [8,9]. Despite these limitations, few alternatives have been developed. Given the high prevalence and disease burden of AD, the need for safe, effective, and affordable topical treatments remains critical.

The historical use of plants and plant-derived substances to reduce inflammation and treat diseases is well documented [10,11,12]. *Citrus unshiu*, cultivated in various regions such as Argentina, Australia, China, Japan, Spain, South Korea, and South Africa, has a long history of medicinal use. A steady stream of research has focused on the therapeutic effects of flavonoids derived from *Citrus unshiu* peel and fruits, particularly naringin [13,14,15].

**Figure 1:**
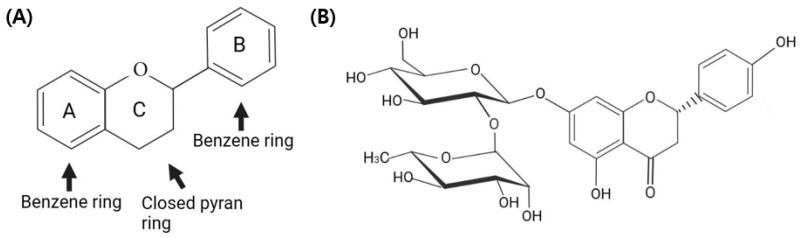
Chemical structures of (**A**) flavonoid and (**B**) naringin.

Naringin, a flavanone glycoside with a molecular formula of C_27_H_32_O_14_ and a specific molecular weight of 272.257 g/mol (Figure 1), has demonstrated potent anti-inflammatory and anti-allergic properties through the modulation of various inflammatory pathways and cytokine production [16,17,18,19]. In addition, naringin exhibits dose-dependent anti-oxidative properties, reducing DNA damage and enhancing antioxidant enzyme activities [20,21].

In this study, we investigated the efficacy and potential mechanisms of topically applied naringin in a mouse model of AD, to address the unmet need for the development of topical treatments for AD.

## 2. Results

**Figure 2:**
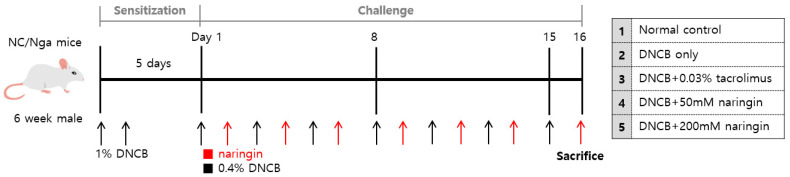
Schematic description of the experiment (n = 6 per group). DNCB, 1-chloro-2,4-dinitrobenzene.

To evaluate the efficacy of naringin in AD, we established an AD mouse model using NC/Nga mice. Sensitization was performed with 1% 1-chloro-2,4-dinitrobenzene (DNCB), followed by the application of 0.4% DNCB three times a week throughout the challenge period to induce AD symptoms, except for the normal control (NC) group (Figure 2).

### 2.1. Naringin Exhibits Clinical Efficacy in Atopic Dermatitis Mouse Model

**Figure 3:**
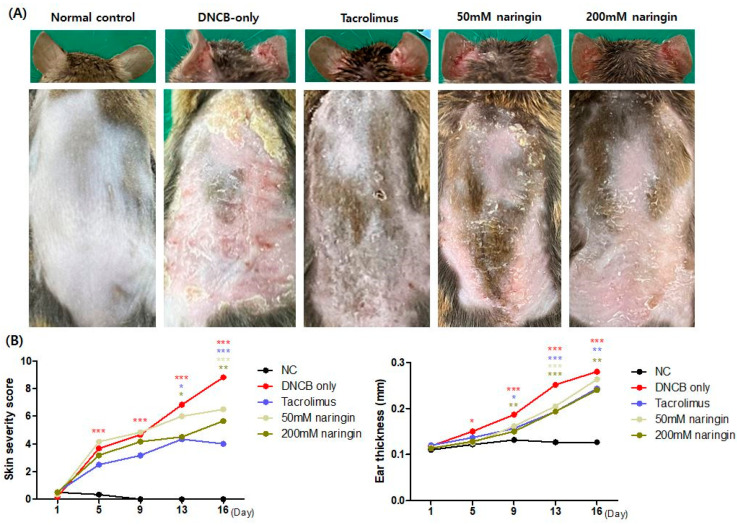
(**A**) Clinical images of the ear and back at the end of the challenge period (day 16). (**B**) Graphs representing dermatitis severity and ear thickness assessment results. Values represent the mean ± SEM (n = 6). Data compared among multiple groups were analyzed using one-way analysis of variance. * *p* < 0.05, ** *p* < 0.01, *** *p* < 0.001 compared to the NC or DNCB-only group. DNCB, 1-chloro-2,4-dinitrobenzene; NC, normal control.

Topical application of naringin significantly improved AD-like clinical symptoms (Figure 3A). Mice in the DNCB group exhibited significantly higher dermatitis severity scores, including erythema/hemorrhage, scaling/dryness, excoriation, and edema, compared to the NC group, confirming the successful induction of AD (Figure 3B, *p* < 0.01). In contrast, naringin treatment significantly reduced these severity scores (Figure 3B, *p* < 0.05). Ear thickness, an indicator of edema, was also significantly lower in the 200 mM naringin group compared to the DNCB-only group (Figure 3B, *p* < 0.01). 

### 2.2. Naringin Exhibits Histopathological Efficacy in Atopic Dermatitis Mouse Model

**Figure 4:**
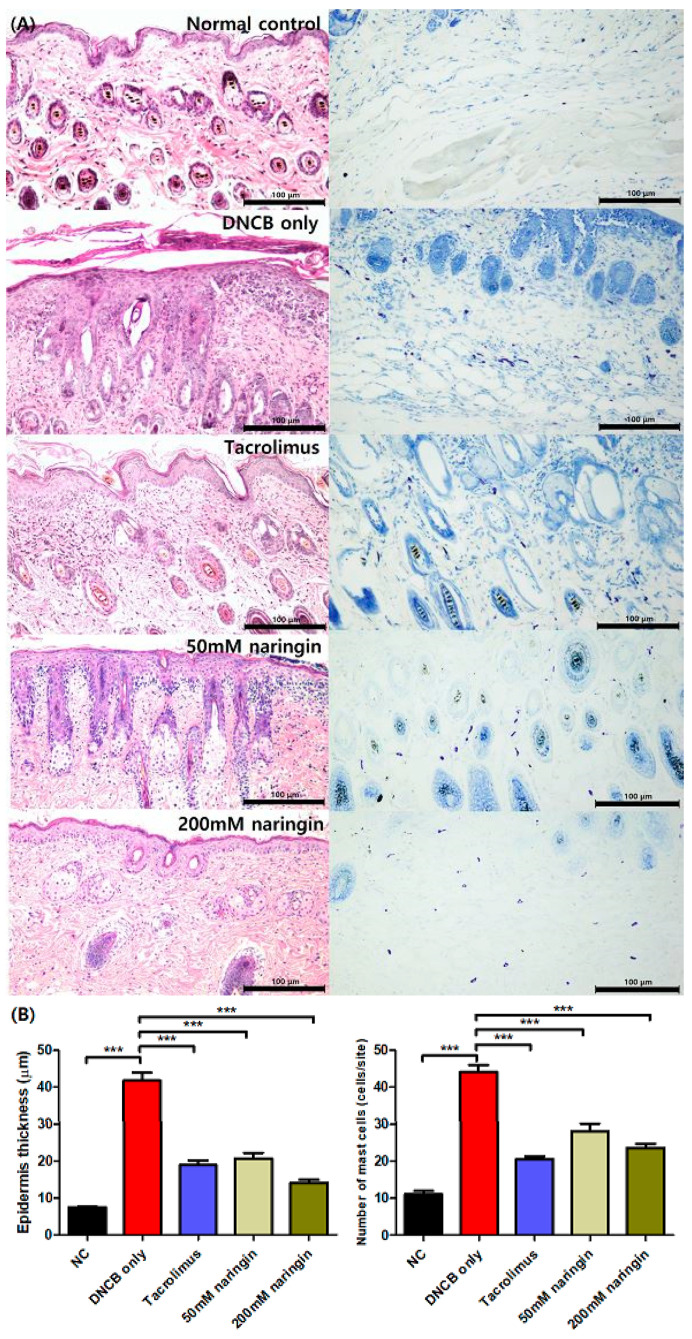
(**A**) Pathology images of H&E and toluidine blue staining in dorsal skin tissue samples. Mast cells are stained purple with toluidine blue. Original magnification = ×200, scale bar = 100 μm. (**B**) Graphs representing the epidermal thickness and the number of mast cells. Values are mean ± SEM (n = 6). Data compared among multiple groups were analyzed using one-way analysis of variance. *** *p* < 0.001 compared to the NC or DNCB-only group. H&E, hematoxylin and eosin; NC, normal control; DNCB, 1-chloro-2,4-dinitrobenzene.

Histopathological analysis further confirmed the efficacy of naringin in suppressing AD-related skin changes (Figure 4). Hematoxylin and eosin (H&E) staining of dorsal skin tissues revealed significantly greater hyperkeratosis and epithelial hyperplasia in the DNCB-only group compared to the NC group, with naringin treatment resulting in a significant reduction in epidermal thickness (Figure 4, *p* < 0.001). Similarly, quantification of mast cells through toluidine blue staining showed a significantly higher number of mast cells in the DNCB-only group, while naringin treatment led to a significant decrease in mast cells (Figure 4, *p* < 0.001).

### 2.3. Naringin Modulates AD-Related Cytokine Changes at the mRNA Level

**Figure 5:**
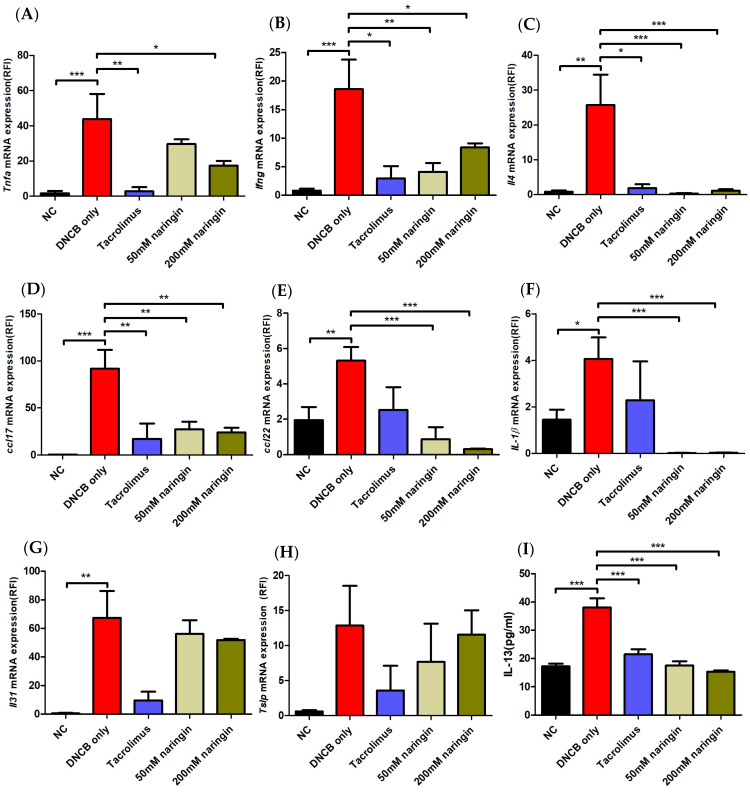
Expressed mRNA level of (**A**) TNF-α, (**B**) IFN-γ, (**C**) IL4, (**D**) CCL17, (**E**) CCL22, (**F**) IL1β, (**G**) IL31, and (**H**) TSLP quantified by qRT-PCR and (**I**) IL13 by ELISA in dorsal skin tissues. The expression of each gene was normalized to that of Actb. Each qRT-PCR reaction was performed in triplicate. Values are mean ± SEM (n = 6). Data compared among multiple groups were analyzed using one-way analysis of variance. * *p* < 0.05, ** *p* < 0.01, *** *p* < 0.001 compared to the NC or DNCB-only group. CCL, chemokine C-C motif ligand; DNCB, 1-chloro-2,4-dinitrobenzene; IL, interleukin; INF-γ, interferon-gamma; NC, normal control; TNF-α, tumor necrosis factor-alpha; TSLP, thymic stromal lymphopoietin.

Molecular analysis of dorsal skin tissues revealed that naringin modulated AD-related cytokine changes are consistent with the clinical and histopathological findings (Figure 5). Quantitative reverse transcription polymerase chain reaction (qRT-PCR) revealed significant decreases in Th2- and Th1-related cytokines, including IL4, thymus and activation-regulated chemokine (TARC)/chemokine C-C motif ligand (CCL) 17, macro-phage-derived chemokine (MDC)/CCL22, IL1β, interferon-gamma (IFN-γ), and tumor necrosis factor-alpha (TNF-α) with naringin treatment (*p* < 0.05). Naringin treatment also led to reductions in IL31 and thymic stromal lymphopoietin (TSLP) compared to the DNCB-only group. Enzyme-Linked Immunosorbent Assay (ELISA) results further confirmed a significant dose-dependent reduction in IL13 levels in naringin-treated groups (*p* < 0.001).

### 2.4. Naringin Alleviates Atopic Dermatitis by Inhibiting the JAK-STAT Pathway

**Figure 6:**
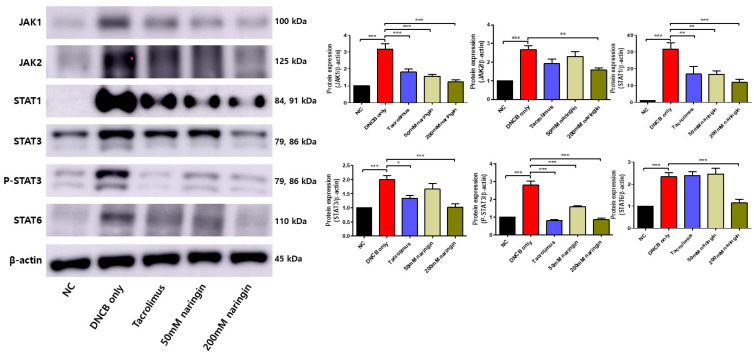
Expression of JAK–STAT proteins in dorsal skin tissues. Immunoblotting intensities were calculated with ImageJ software (version 1.8.0). Values are mean ± SEM (n = 6). Data compared among multiple groups were analyzed using one-way analysis of variance. * *p* < 0.05, ** *p* < 0.01, *** *p* < 0.001 compared to the NC or DNCB-only group. DNCB, 1-chloro-2,4-dinitrobenzene; JAK-STAT, Janus kinase-signal transducer and activator of transcription; NC, normal control; P-STAT3, phospho-STAT3.

Western blot analysis demonstrated that naringin modulates the expression of proteins involved in the Janus kinase (JAK)-signal transducer and activator of transcription (STAT) pathway in the AD mouse model (Figure 6). The DNCB-only group showed significantly increased expression of JAK1, JAK2, STAT1, STAT3, phospho-STAT3 (p-STAT3), and STAT6 compared to the NC group (Figure 6, *p* < 0.001). Naringin treatment resulted in a statistically significant downregulation of JAK1, JAK2, STAT1, STAT3, p-STAT3, and STAT6 in a dose-dependent manner (Figure 6, *p* < 0.05).

## 3. Discussion

This study investigated the efficacy of topical naringin using an AD mouse model induced by DNCB. The DNCB-only group exhibited significantly increased dermatitis scores, ear and epidermal thickness, and mast cell count (Figure 3 and Figure 4), with elevated mRNA levels of pro-inflammatory cytokines, including TNF-α and IL1β, as well as Th1-related cytokines like IFN-γ and Th2-related cytokines such as IL4, IL13, IL31, TSLP, TARC/CCL17, and MDC/CCL22 (Figure 5). DNCB acts as an allergen-associated hapten that activates local skin dendritic cells, triggering Th cell responses and leading to inflammatory responses that mimic key features of human AD [22,23]. The observed findings in the DNCB-only group confirm that the mouse model in this study effectively replicates the clinical, histological, and immunological features of AD.

In this study, topical naringin significantly reduced dermatitis severity and skin thickness, representing alleviated skin inflammation—edema, vascular permeability, inflammatory cell infiltration, and epidermal hyperplasia (Figure 3). During inflammatory responses in the skin, the activation of nuclear factor kappa B (NF-κB) signaling is crucial both at the initiation and throughout the progression of inflammation, inducing pro-inflammatory cytokines such as TNF-α, IL1, and IL6, adhesion molecules, chemokines, and enzymes such as cyclooxygenase (COX)-2 [24,25,26]. COX enzymes catalyze the conversion of arachidonic acid to prostaglandins, and COX-2 is recognized for its role in various inflammatory diseases, including skin inflammation, allergic asthma, and rheumatoid arthritis. [27,28] COX-2 is produced in response to inflammatory signals, physiological stimuli, and growth factors and plays a key role in the generation of prostaglandins, which contribute to pain and promote inflammation [29]. Previous studies reporting on naringin’s protective and regenerative effects on skin have noted its anti-inflammatory properties through the reduction in NF-κB and COX-2 expression [30,31]. CX3C motif chemokine receptor (CX3CR) 1 macrophages are important in the inflammatory response and release TNF and TGFβ1. These factors stimulate hair follicle stem cells (HFSCs), leading to wound-induced hair follicle neogenesis and hair follicle regeneration [32,33,34,35]. On the other hand, Langerhans cells, suprabasal HFSCs, sebaceous gland filling and the surrounding microbiome are also involved in the wound response, the process of which may interact with our findings [36,37,38]. Besides, naringin demonstrated significant antioxidant properties by increasing glutathione levels and superoxide dismutase activity, thereby inhibiting reactive oxygen species (ROS). Moreover, naringin has been shown to reduce DNA damage, oxidative stress, and various inflammatory markers, including TNF-α, total leukocytes, neutrophils, tissue edema, and inflammatory cell infiltration [20,21].

Our results revealed that topical naringin significantly reduced the number of mast cells in AD skin lesions (Figure 4). Mast cells play a crucial role in inflammatory responses by releasing potent signaling molecules like histamine, which intensifies inflammation and contributes to symptoms such as erythema, swelling, and pruritus [39]. Histamine is a major driver of the severe itching commonly experienced by AD patients. Additionally, mast cells release IL4 and IL13, amplifying the Th2 immune response, a hallmark of the immunological profile of AD. Previous studies have shown that naringin inhibits histamine release from mast cells in vitro, as well as histamine, inflammatory cytokines, and immunoglobulin (Ig) E in vivo [40,41]. Furthermore, the oral administration of naringin in an oxazolone-induced AD mouse model dose-dependently suppressed both type 1 and type 4 skin allergic reactions [42]. Our findings suggest that naringin alleviates AD symptoms by modulating mast cell infiltration and reducing the release of inflammatory mediators, contributing to its therapeutic effects beyond mere histamine inhibition.

In this study, the DNCB-only group exhibited a significant increase in the expression of IL4, IL13, IL31, TSLP, TARC/CCL17, MDC/CCL22, IFN-γ, IL1β, and TNF-α, all of which are consistently expressed with AD. Naringin treatment significantly reduced characteristic cytokines of AD including IL4, IL13, TARC/CCL17, MDC/CCL22, IFN-γ, IL1β, and TNF-α (Figure 5). IL4 and IL13 are key biomarkers in AD pathogenesis, driving the Th2 immune responses. IL31, which is correlated with the IL4 and IL13 expressions, is another Th2 cytokine. They contribute to hallmark features such as pruritus and barrier dysfunction [43,44,45,46,47,48]. IFN-γ and TNF-α, primarily secreted by Th1 cells, are more prominent in the chronic phase of AD and exacerbate inflammation [49,50]. IFN-γ, in conjunction with Th2 cytokines, promotes macrophage activation, increases TSLP secretion, and alters stratum corneum lipid composition, contributing to the chronicity of AD [51]. Furthermore, IFN-γ has been shown to downregulate mRNA levels of key enzymes involved in ceramide synthesis in three-dimensional epidermal models, while TNF-α, either alone or in combination with Th2 cytokines, decreased levels of long-chain free fatty acids and ceramides, both of which compromise the skin barrier [51,52,53]. Naringin’s ability to reduce these cytokines suggests it may not only alleviate inflammation but also help restore the skin barrier, offering a dual benefit in treating AD.

TARC/CCL17 and MDC/CCL22 are chemokines that recruit immune cells to inflamed tissues and have emerged as biomarkers for AD, with a correlation to clinical severity [54,55,56,57,58,59]. These established biomarkers are often used as reliable tools to predict clinical prognosis and therapeutic outcomes, enabling more personalized and effective treatment strategies for patients. TARC/CCL17, a Th2 chemoattractant, has been recognized as a prognostic biomarker for AD [60,61,62,63]. In Japan, TARC/CCL17 has been measured under health insurance coverage for monitoring treatment effectiveness since 2008 [61]. Activation of the IL22/IL22Rα pathway in AD induces TARC/CCL17, IL1α, and IL6, recruiting CCR4+ T cells. In severe AD, regulatory T cells (Tregs) with high CCR4 expression are recruited but show reduced ability to secrete TGF-β and IL10, indicating impaired Treg function in AD [64]. On the other hand, lesional MDC/CCL22 level has been found as a promising predictive biomarker for AD [59,65,66]. MDC/CCL22 has been used across multiple studies validating treatment responses of topical crisaborole, oral cyclosporine, and a targeted treatment, fezakinumab [6,54,67]. Naringin’s ability to reduce the expression of these key biomarkers suggests that naringin can be utilized for the treatment of AD.

The JAK-STAT signaling pathway significantly regulates Th2 differentiation in AD, with elevated levels of STAT proteins associated with disease severity [48,68]. Naringin was found to reduce the expression of STAT1, STAT2, STAT3, phosphorylated STAT3 (P-STAT3), STAT6, JAK1, and JAK2, which are key components of this pathway (Figure 6). This suggests that naringin may alleviate AD by modulating the JAK-STAT signaling pathway. JAK1 and JAK2 are mediated by IL31 to activate IL31RA, leading to STAT3-mediated β-endorphin production, potentially contributing to peripheral itching in AD [69]. IL-4 engages two types of receptors. It binds to type 1 receptors (IL-4Rα and γ chains), which activates JAK1 and JAK3, leading to STAT6 activation. Conversely, IL-4 and IL-13 interact with type 2 receptors (IL-4Rα and IL-13Rα1), activating JAK1 and TYK2, which subsequently activate STAT6 and STAT3 [70,71]. STAT1, STAT2, and STAT3 play a role in mediating pro-inflammatory signals. At the same time, STAT6 is essential for Th2 signaling [72,73]. Additionally, the signaling pathways of TSLP and IL31 also involve JAK1 and JAK2, impacting STAT1, STAT3, and STAT5 [74,75]. Inhibiting these pathways has been shown to reduce inflammation and improve clinical outcomes in AD [76,77,78]. This extends to topical applications as well, with ruxolitinib cream, a selective JAK1/JAK2 inhibitor, receiving the Food and Drug Administration (FDA) approval for the treatment of AD, and momelotinib, another JAK1/JAK2 inhibitor, demonstrating significant anti-inflammatory effects through topical application [79,80]. Naringin’s ability to reduce the expression of JAK1, JAK2, and various STAT proteins suggests that it interferes with the signaling cascades that drive inflammation in AD, reducing inflammation and improving skin condition.

While this study highlights the efficacy of naringin in modulating inflammatory responses in the skin lesions of AD, further analysis of Th2 cells and type 2 innate lymphoid cells (ILC2s) as key drivers of the pathology is needed. In addition, we used only male mice in our experiments to prevent hormonal fluctuations from influencing inflammatory and immune responses, which may limit the generalizability of our findings. Further investigation is warranted to validate whether naringin alters the phenotype of immune cells, thereby clarifying its mechanism of action, as well as to examine potential differences in efficacy across different sexes.

## 4. Materials and Methods

### 4.1. Animal Study

Specific pathogen-free male NC/Nga mice, 4 weeks old and weighing between 16–21 g, were obtained from Central Laboratory Animals Inc. (Seoul, Republic of Korea). The mice were quarantined for one week and acclimated for another one week; the mice were 6 weeks old at the beginning of the experiment. A total of 30 mice were used, with 6 mice assigned to each group. The animals were housed in a temperature-controlled environment (23 ± 3 °C) with 50 ± 10% relative humidity on a 12-h light/dark cycle. Water and commercial rodent chow were provided ad libitum.

### 4.2. Atopic Dermatitis Mouse Model and Drug Challenge

The mice were divided into five groups as follows: normal control (NC; n = 6), DNCB-only (DNCB, Sigma-Aldrich, St Louis, MO, USA; n = 6), Tacrolimus (DNCB + 0.03% tacrolimus ointment, Protopic^®^, LEO Pharma, Ballerup, Denmark; n = 6), and 50 mM/200 mM naringin (DNCB + naringin, Sigma-Aldrich, St Louis, MO, USA; n = 6 per group). Figure 2 schematically represents the experimental schedule. The DNCB solution was prepared at a concentration of 1% and 0.4% in the vehicle, which consisted of a glycerol/ethanol suspension (1:4). For sensitization, 1% DNCB was applied to the abdomen of mice twice a week, except the NC group. After the sensitization, the dorsal hair of mice was shaved using animal clippers and hair removal cream followed by 48 h of stabilization. During the 16-day challenge period, 0.4% DNCB was applied to the mice, except for the NC group, thrice a week. The mice in each group were applied with 0.03% tacrolimus ointment, 50 mM naringin, or 200 mM naringin thrice a week as well. Topical tacrolimus, one of the topical treatments recommended by AD treatment guidelines, was used as a standard of care control. The concentrations of naringin were selected based on our preliminary in vivo experiments with 10 mM, 50 mM, and 100 mM topical naringin, the concentrations of which were based on previous studies [30,31]. The volume of DNCB, vehicle solution, and naringin applied to each group was 50 μL for the back and 20 μL for each ear. On day 16, dorsal and both ear skin tissues were collected 4 h after the last application. All animal research procedures were conducted in compliance with the Laboratory Animals Welfare Act, the Guide for the Care and Use of Laboratory Animals, and the Guidelines and Policies for Rodent Experiments established by the Institutional Animal Care and Use Committee of the School of Medicine, The Catholic University of Korea (CUMS-2023-0145-01).

### 4.3. Dermatitis Severity and Ear Thickness Assessment

Ear thickness was assessed twice weekly using a digital caliper (Kroeplin, Schlüchtern, Germany). Dermatitis severity was scored by two blinded investigators, based on clinical photographs taken twice weekly, and the average was calculated; each mouse was scored on a scale of 0–3 for erythema/hemorrhage, scaling/dryness, excoriation, and edema. The final score was defined as the sum of these individual scores (maximum score, 12).

### 4.4. Histological Assessment

Dorsal skin tissues were fixed in 10% neutral buffered formalin for histological analysis and were then paraffin-embedded. The tissues were sectioned at 4 μm thickness and stained with H&E to analyze histological changes. Epidermal thickness was measured in three randomly selected areas, with five points randomly selected in each area on the slides strained with H&E using a microscope (Leica, Hessen, Germany). Mast cell count in dorsal skin tissues was evaluated by toluidine blue staining, and the number of mast cells was counted in three randomly selected areas. All measurements were performed using ImageJ Fiji software (Version 1.54 g; WS Rasband, National Institute of Health, Bethesda, MD, USA).

### 4.5. Quantitative Real-Time PCR

Total RNA was isolated from each sample using TRIzol reagent (Invitrogen, Carlsbad, CA, USA) according to the manufacturer’s guidelines. RNA concentration and purity (260:280 ratio) were determined using a NanoDrop spectrophotometer (Thermo Fisher Scientific, Waltham, MA, USA). For qRT-PCR, the synthesized primers were diluted and mixed with Power SYBR^®^ Green PCR Master Mix (Takara Biomedical Inc., Shiga, Japan). The primer sequences used in this study are provided in Table 1. Quantitative real-time PCR (qRT-PCR) was performed on a CFX-96 thermocycler (Bio-Rad, Hercules, CA, USA), with an initial denaturation at 95 °C for 10 min, followed by 45 cycles of 95 °C for 15 s and 60 °C for 30 s. Gene expression levels were calculated using the cycle threshold (Ct) values and the ΔCt method. Each qRT-PCR reaction was performed in triplicate. Data were normalized to the housekeeping gene β-actin and presented as a ratio relative to the untreated control.

### 4.6. ELISA Assay

The levels of IL13 in the dorsal skin tissues of the models were quantified using ELISA kits (Labiskoma, Seoul, Republic of Korea) as per the manufacturer’s guidelines. Data were obtained using a microplate reader (VersaMax, Molecular devices, Silicon Valley, CA, USA).

### 4.7. Western Blot

Protein lysates were extracted using T-PER lysis buffer (Thermo Fisher Scientific, Waltham, MA, USA) supplemented with a protease inhibitor cocktail (Thermo Fisher Scientific, Waltham, MA, USA). Protein concentrations were measured using a BCA Protein Assay Kit II (Thermo Fisher Scientific, Waltham, MA, USA). Equal amounts of protein (20 or 40 μg) were loaded onto 6–15% SDS-PAGE gels, separated, and transferred onto polyvinylidene fluoride membranes (MilliporeSigma, St. Louis, MO, USA). After blocking with either 5% skim milk or 5% BSA in TBS-T (Tris-buffered saline with 0.1% Tween 20), membranes were incubated overnight at 4 °C with primary antibodies diluted in 5% BSA/TBS-T. The following primary antibodies were used: mouse monoclonal anti-β-actin (1:2500, #3700), JAK1 (1:400, #3344), JAK2 (1:1000, #3230), STAT1 (1:1000, #14994), STAT3 (1:1000, #9139), anti-P-STAT3 (1:1000, #9145), and STAT6 (1:1000 #9362S). After incubation, membranes were washed four times with TBS-T and treated with horseradish peroxidase-conjugated goat anti-mouse or rabbit IgG secondary antibodies (GTX213111-01 or GTX213110-01; GeneTex, Irvine, CA, USA) for two hours at room temperature. Following another set of four washes in TBS-T, protein bands were visualized using an ECL substrate (Thermo Fisher Scientific, Waltham, MA, USA) and captured with an Amersham™ Imager 600 (GE Healthcare, Chicago, IL, USA). The band intensities were analyzed using ImageJ software (version 1.8.0) (U.S. NIH, Bethesda, MD, USA).

### 4.8. Statistics Analysis

Statistical analysis was conducted using one-way analysis of variance (ANOVA) followed by Tukey’s multiple comparisons test. For comparisons between two groups, unpaired *t*-tests were employed. Graphs were generated using GraphPad Prism 5 (GraphPad Software, La Jolla, CA, USA), and all data are expressed as mean ± standard error of the mean (SEM). Statistical significance was set at *p* < 0.05 (* *p* < 0.05; ** *p* < 0.01; and *** *p* < 0.001).

## 5. Conclusions

Based on the results of our study, the topical application of naringin may effectively alleviate the symptoms of AD, primarily through its anti-inflammatory properties. These findings suggest that naringin could serve as a therapeutic agent for AD.

## Figures and Tables

**Table 1:** Primer sequences for quantitative real-time PCR amplifications.

Target Gene	Forward/Reverse	Sequence (5′-3′)
*Il4*	F	TCACTGACGGCACAGAGCTA
	R	CTTCTCCTGTGACCTCGTT
*Il31*	F	ACACCGAGTTGGAGAGCCGTAT
	R	CTGTCCTCAGACCGATGTTCTC
*Tarc*/*Ccl17*	F	CGAGAGTGCTGCCTGGATTACT
	R	GGTCTGCACAGATGAGCTTGCC
*Mdc*/*Ccl22*	F	GTGGAAGACAGTATCTGCTGCC
	R	AGGCTTGCGGCAGGATTTTGAG
*Tslp*	F	AGCTTGTCTCCTGAAAATCGAG
	R	AGGTTTGATTCAGGCAGATGTT
*Tnfa*	F	AACTCCAGGCGGTGCCTATG
	R	TCCAGCTGCTCCTCCACTTG
*Ifng*	F	AAGCGTCATTGAATCACACC
	R	TGACCTCAAACTTGGCAATA
*Il1b*	F	TGGACCTTCCAGGATGAGGACA
	R	GTTCATCTCGGAGCCTGTAGTG
*Actb*	F	CATTGCTGACAGGATGCAGAAGG
	R	TGCTGGAAGGTGGACAGTGAGG

*Ccl*, chemokine C-C motif ligand; *Il*, interleukin; *Infg*, interferon-gamma; *Mdc*, macrophage-derived chemokine; *Tarc*, thymus and activation-regulated chemokine; *Tnfa*, tumor necrosis factor-alpha; *Tslp*, thymic stromal lymphopoietin.

## Data Availability

The original contributions presented in the study are included in the article, further inquiries can be directed to the corresponding author.
